# Reduced cerebral blood flow and oxygen metabolism in extremely preterm neonates with low-grade germinal matrix- intraventricular hemorrhage

**DOI:** 10.1038/srep25903

**Published:** 2016-05-16

**Authors:** Pei-Yi Lin, Katherine Hagan, Angela Fenoglio, P. Ellen Grant, Maria Angela Franceschini

**Affiliations:** 1 Athinoula A. Martinos Center for Biomedical Imaging, Massachusetts General Hospital/Harvard Medical School, Charlestown, MA 02129, USA; 2 Institute of Child Development, University of Minnesota, Minneapolis, MN 55455, USA; 3 Fetal-Neonatal Neuroimaging and Developmental Science Center, Boston Children’s Hospital/Harvard Medical School, Boston, MA 02115, USA

## Abstract

Low-grade germinal matrix-intraventricular hemorrhage (GM-IVH) is the most common complication in extremely premature neonates. The occurrence of GM-IVH is highly associated with hemodynamic instability in the premature brain, yet the long-term impact of low-grade GM-IVH on cerebral blood flow and neuronal health have not been fully investigated. We used an innovative combination of frequency-domain near infrared spectroscopy and diffuse correlation spectroscopy (FDNIRS-DCS) to measure cerebral oxygen saturation (SO_2_) and an index of cerebral blood flow (CBF_i_) at the infant’s bedside and compute an index of cerebral oxygen metabolism (CMRO_2i_). We enrolled twenty extremely low gestational age (ELGA) neonates (seven with low-grade GM-IVH) and monitored them weekly until they reached full-term equivalent age. During their hospital stay, we observed consistently lower CBF_i_ and CMRO_2i_ in ELGA neonates with low-grade GM-IVH compared to neonates without hemorrhages. Furthermore, lower CBF_i_ and CMRO_2i_ in the former group persists even after the resolution of the hemorrhage. In contrast, SO_2_ does not differ between groups. Thus, CBF_i_ and CMRO_2i_ may have better sensitivity than SO_2_ in detecting GM-IVH-related effects on infant brain development. FDNIRS-DCS methods may have clinical benefit for monitoring the evolution of GM-IVH, evaluating treatment response, and potentially predicting neurodevelopmental outcome.

Twelve percent of live births in the United States are premature, resulting in the need to care for some 500,000 premature infants every year^1^. This rate is 30 percent greater than it was in the 1980s, and the National Academies estimate that premature births cost the US in excess of $26 billion annually^2^. Germinal matrix-intraventricular hemorrhage (GM-IVH) is a major complication of prematurity, occurring in about 45 percent of extremely low birth weight infants (weight < 1000 g)^3^. Even as improved neonatal intensive care and technological advances have increased the survival rate of extremely premature infants, the high incidence of GM-IVH has remained unchanged over the past decade^4^.

About 50–80 percent of premature infants with GM-IVH are diagnosed as low-grade (Grade I and II), and the majority of these newborns are estimated to be asymptomatic^5^,^6^. Diagnosis of GM-IVH in premature infants largely relies on screening via head ultrasound (HUS), even though the reliability in grading mild hemorrhage by HUS is low^7^,^8^. Unlike premature infants with severe GM-IVH, the relationship of low-grade GM-IVH to adverse outcomes is less predictable^9^,^10^. Moreover, current neuroimaging tools are not adequate surrogates for long-term neurodevelopmental follow-up. Major HUS abnormalities are only moderately predictive of cerebral palsy (CP) at variable ages of follow-up^11^,^12^,^13^,^14^,^15^ with still less predictive power where cognitive delay is concerned^14^,^15^. Meanwhile, magnetic resonance imaging (MRI) scanning as a predictive tool is still an area of active research^16^. Thus, there is a need for the discovery of new biomarkers of cerebral health in extremely premature infants. Such biomarkers may ultimately aid in the early detection and/or prevention of low-grade GM-IVH and may similarly have prognostic application.

Immature cerebral vascular anatomy and hemodynamic regulation in premature infants directly contributes to the pathogenesis of GM-IVH. Fluctuations in cerebral blood flow (CBF) associated with prematurity put the highly vascularized germinal matrix (GM) at risk for hemorrhage^17^,^18^, In full-term infants the GM regresses before birth^19^, but in infants born significantly pre-term this is not the case. The special vascular morphology of the GM serves to meet the significant metabolic demands of a wave of cell proliferation and migration from the GM to the cortex prior to term gestational age^19^. Autopsies of premature infants find hemorrhages in the GM are associated with decreased neuronal migration to the cortex^20^. The reduction in brain cell proliferation caused by germinal matrix hemorrhage results in a reduction of cerebral myelination^21^. Thus, although the GM is a transitory deep brain structure, hemorrhage in the GM has the potential to impair normal cortical development in affected infants^22^. GM-IVH may therefore have an observable impact on cortical physiology.

Cerebral hemoglobin oxygen saturation (SO_2_) is readily measured by near-infrared spectroscopy-based (NIRS) cerebral oximeters. However, the clinical utility of SO_2_ is often inconclusive^23^,^24^ since changes in SO_2_ reflect changes in both cerebral oxygen consumption and systemic changes in oxygen supply^25^. To disentangle the contributions of oxygen supply and demand, cerebral blood flow (CBF) must be measured in order to determine an unambiguous cerebral metabolic rate.

Frequency domain NIRS combined with diffuse correlation spectroscopy (FDNIRS-DCS) is a new technology for non-invasive measurement of cerebral blood flow and metabolic oxygen consumption (CMRO_2_). DCS measures an index of CBF (CBF_i_) in units of cm^2^/s. Although the units of CBF_i_ are unconventional, CBF_i_ values have good agreement with absolute CBF measured by other modalities^26^,^27^,^28^. Due to the difference in units, the subscript *i* is used to distinguish DCS-measured values from the physiological parameters they estimate. From CBF_i_ and FDNIRS measurements of absolute cerebral hemoglobin concentrations, an estimate of oxygen consumption (CMRO_2i_) is calculated^29^. CBF_i_ and CMRO_2i_ from FDNIRS-DCS measurements have been reported to be more sensitive to detect early brain growth in infants than the more commonly measured SO_2_. CBF_i_ and CMRO_2i_ increase with postmenstrual age (PMA) and can detect differences in brain maturation in neonates^30^,^31^. Moreover, FDNIRS-DCS-measured CMRO_2i_ has been used to assess individual treatment response in neonates with hypoxic ischemic encephalopathy^32^ and congenital heart disease^33^. In this study, we performed FDNIRS-DCS measurements in extremely low gestational age (ELGA) neonates with and without low-grade GM-IVH on a weekly basis during their hospital stay up until discharge at full-term age. We hypothesized that lower CBF and CMRO_2_ would be detected and sustained in neonates with low-grade GM-IVH in the first few months of life.

## Results

Table 1 summarizes the clinical characteristics of infants in both GM-IVH and control groups. There were no significant inter-group differences with regard to other complications due to prematurity. Premature infants in the GM-IVH group had younger gestational ages at birth than the control group; however, the postmenstrual age (PMA), which is more closely correlated with changes in CBF and CMRO_2_ during early development^31^, was the same for both groups for each measurement session.

Among seven GM-IVH infants, two infants had unilateral grade I GM-IVH and one infant had unilateral grade II. The remaining infants had bilateral GM-IVH with asymmetrical severity. Six infants were identified as having GM-IVH on the first HUS (Day of Life 1–3) and one infant’s diagnosis was made on the second HUS on the sixth day of life. In six of the seven infants, HUS records characterized the hemorrhage as resolved at Day of Life 30, while one infant’s hemorrhage was found to be resolved at Day 60.

**Figure 1:**
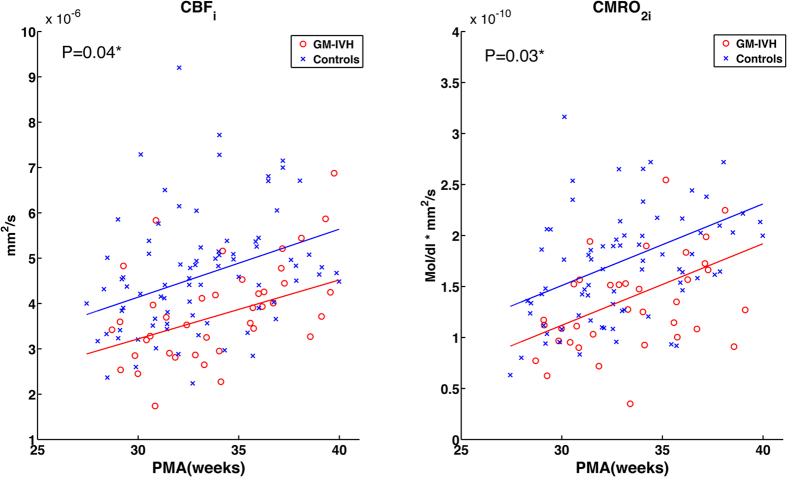
Scatterplots and estimated time trajectories (solid line) of CBF_i_ and CMRO_2i_ with PMA in premature infants with low-grade GM-IVH (red line) and controls (blue line). *Indicates significant inter-group difference (P < 0.05).

**Figure 2:**
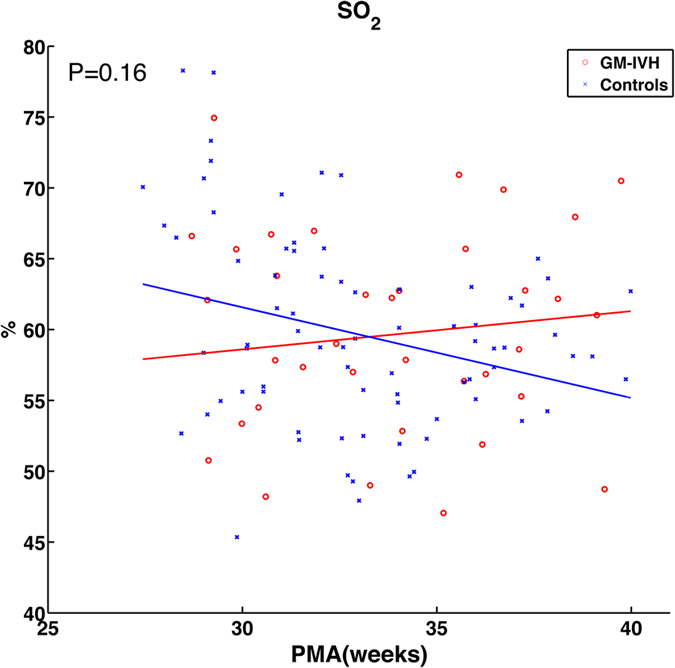
Scatterplots and estimated time trajectories (solid line) of CBF_i_ and CMRO_2i_ with PMA in premature infants with low-grade GM-IVH (red line) and controls (blue line).

Figure 1 shows CBF_i_ and CMRO_2i,_ as a function of PMA. Slopes of the observed measures were modeled from data taken between 27–40 weeks PMA, the ages at which most of the subjects in the two groups were measured. The CBF_i_ and CMRO_2i_ in both groups increased with time but were statistically lower in the GM-IVH group than in the control group (P = 0.04 and 0.03, respectively). Figure 2 presents the weekly average of SO_2_ as a function of PMA with standard errors. Unlike CBF_i_ and CMRO_2i,_ SO_2_ did not show a significant correlation with PMA or a significant difference between groups (P = 0.16).

## Discussion

This study demonstrated that the ELGA infants with low-grade GM-IVH had lower CBF and CMRO_2_ than PMA-matched controls, and this difference persists even after the hemorrhages appeared resolved on HUS images. The findings suggest that low-grade GM-IVH in ELGA infants may have a more significant impact on brain development than is currently assumed.

Our study is the first to demonstrate that mild hemorrhage reduces cerebral metabolism in ELGA infants, and this lowered cerebral metabolism is consistent with the hypothesis that GM-IVH decreases neuronal migration to the cortex^20^. The ganglionic eminence (GE) is a transitory brain region which is the source of critical waves of cell proliferation and migration to the cortex, including a class of neocortical GABAergic interneurons^34^ as well as neurons for the basal nuclei^35^. The GE regresses gradually^19^ with final involution of the GE by 34–36 weeks^36^. Thus, infants are born prematurely retain the GE through birth and experience a portion of this involution *ex utero.* Unfortunately, extremely premature infants with immature hemodynamic regulation often put the highly vascularized GE at risk of rupture leading to a germinal matrix hemorrhage. Thus, if the hemorrhage arises from the GE, it is presumed to affect the production of GABAergic interneurons for the neocortex and the coordination and integration of cortical functions^22^. Del Bigio has reported significant and persistent suppression of cell proliferation following hemorrhage in human GE samples compared to brains without GM-IVH^20^. There is also no evidence of rebound cell proliferation above normal levels in these GM-IVH brains, suggesting the GE continues to involute following hemorrhages without compensation. Another imaging study using MRI to measure cortical volume in premature infants reported reduced cortical volume in low-grade GM-IVH cases at ages coinciding with those in our study^37^. Together, these results suggest that small hemorrhages within the germinal matrix at early stages of gestation may have substantial effects on cortical development in the brains of premature infants.

In contrast to previous studies reported in the literature, which mainly examine the impact of severe GM-IVH at an acute stage, we have instead focused on low-grade GM-IVH and have extended the window of observations to cover longer periods, collecting measurements even after hemorrhages were deemed resolved under HUS examination. Because cerebral blood flow and cerebral oxygen metabolism are persistently lower in GM-IVH infants, we hypothesize that the impact of mild GM-IVH may be of clinical significance and associated with poorer neurodevelopmental outcomes^9^,^11^,^38^,^39^. Indeed, several large retrospective cohort studies report premature infants with low-grade GM-IVH go on at later ages to experience higher rates of CP^9^,^39^, major neurologic abnormality^39^,^40^, and moderate to severe neurosensory impairment^38^. Although other studies posit that such correlations may be a consequence of clinical practices at birth^41^, the higher risks of poor developmental outcomes in premature infants with low-grade GM-IVH suggest that GM-IVH itself may place development of the immature brain at additional risk.

In addition to reduced cerebral metabolism secondary to the presence of hemorrhage, low cerebral blood flow itself may also be a causative factor for the onset of GM-IVH^42^,^43^. Prior to our work, indirect measures of CBF fluctuations had been frequently observed before and/or immediately after the hemorrhage occurred in GM-IVH neonates by computed tomography (CT)^44^, positron emission tomography (PET)^45^, conventional NIRS^46^, and Doppler ultrasound on superior vena cava flow^47^, but these studies were restricted to measurements during the first few days of life. These findings from these other imaging modalities are consistent with the hypothesis that the ischemia-reperfusion cycle drives the pathogenesis of IVH^48^,^49^. Our method equips future prospective studies to investigate further the role of impaired CBF regulation in the risk for developing IVH.

Few studies have reported cerebral perfusion or function in preterm infants with mild or uncomplicated GM-IVH and the association of perfusion and function with infants’ later outcomes^50^. A case-control study using conventional NIRS methods for two hours a day for eight days found persistently lower cerebral rSO_2_ and higher cerebral oxygen extraction fraction (COEF) in preterm neonates with GM-IVH^46^. It was suspected that the higher COEF observed in infants with low-grade GM-IVH resulted from low CBF. Increased COEF was also observed after severe hemorrhages occurred and the magnitude of the increase was identical to that observed in lower-grade cases. In the same cohort of infants with low-grade GM-IVH, it was later reported that high and low SO_2_ on the first day of life was associated with poorer cognitive outcomes among preterm infants at 2 to 3 years old^50^. This further demonstrates the clinical value of monitoring cerebral physiology in the early postnatal period to predict long-term outcomes^51^. In the current work, our novel combination of DCS with FDNIRS found that lower CBF_**i**_ and CMRO_**2i**_ were consistently observed in premature neonates with GM-IVH throughout the first few months of life, while SO_2_ did not show a significant difference between affected and unaffected infants. Our results suggest that CBF_i_ and CMRO_2i_ are more robust measures than SO_2_ for investigating the effects of mild hemorrhages on cortical development and thus may have an important role as an early outcome predictor in this population.

Our results also demonstrate that bedside measures of cerebral blood flow and metabolism measured with FDNIRS-DCS have the potential to detect early abnormal cerebral physiology in preterm infants. Previous NIRS studies using commercial continuous-wave NIRS (CWNIRS) systems use optical absorption to measure changes in hemoglobin concentration and, with some assumptions, estimate SO_2_ and COEF – a standard measure of oxygen delivery from capillaries to tissue. In contrast, the FDNIRS used in this study measures both the optical absorption and the phase-shift of modulated light to determine separate scattering and absorption coefficients of tissue. Thus, FDNIRS is superior in quantifying absolute cerebral hemoglobin concentrations, eliminating some assumptions required with CWNIRS^52^. However, the interpretation of COEF values by either method alone is not always straightforward because changes in the COEF represent differences in *either* oxygen supply *or* consumption. In order to correctly account for the distinct contributions of supply and demand, we directly measure cerebral blood flow independently so we can unambiguously determine metabolic rate, especially under pathological conditions^25^. Our FDNIRS-DCS method provides a direct measure of CBF and CMRO_2,_ a more direct measure of neuronal health. Our results also demonstrated that SO_2_ is not significantly different between the two groups, which is in agreement with our previous findings of smaller variance in SO_2_ with age and disease than in CBF_**i**_, and CMRO_**2i**_^25^,^53^. Thus, CBF_i_ and CMRO_2i_ detect the impact of GM-IVH more effectively than SO_2_ alone, and potentially may be a better predictor of outcomes.

The premature neonates in this study did not undergo any clinical MRIs during their hospital stay. Some subtle injuries associated with white matter, brain stem, and cerebellar hemorrhage may not be detected by ultrasound imaging. However, the effects of subtle injuries in cerebral hemodynamics and metabolism have not been fully characterized, and even premature infants without GM-IVH may have a similar chance of hemorrhage occurring in other areas of the brain.

Due to the relatively small sample of infants in this study, there is insufficient statistical power to examine hemispheric differences in hemodynamics. Previously, COEF measured at the left fronto-parietal side showed no statistical difference among infants with unilateral and bilateral GM-IVH^46^, suggesting hemorrhage-related alterations in CBF may occur globally. On the other hand, Ment *et al*. found higher blood flow in the side on which the hemorrhages appeared, but these hemisphere differences were only observed in first five days of life^54^. We plan to conduct larger cohort studies in the future using the FDNIR-DCS method to investigate whether hemispheric differences exist among GM-IVH-associated changes of CBF and/or CMRO_2._ Our ultimate aim is to identify adequate biomarkers that are predictive of long-term neurodevelopmental outcomes.

## Conclusion

Our results demonstrated low-grade GM-IVH-related changes in CBF_i_ and CMRO_2i_ in ELGA infants. Our new technology makes quantification of CBF_i_ and CMRO_2i_ feasible right at the bedside, without the need for radioactive tracers, and with a sensitivity to mild GM-IVH cases that is lacking in existing measures. This method makes it possible for the first time to follow changes in cerebral cortical metabolism as neuronal migration progresses from the GM to the cortex in extremely low gestational age neonates.

## Methods

The study protocol was reviewed and approved by the Institutional Review Board for Partners Healthcare, the Partners Human Research Committee (PHRC). The study method was designed and carried out in accordance with PHRC requirements and the regulations that govern human subjects research.

### Participants

Seven ELGA infants with low-grade GM-IVH and a control group of 13 ELGA infants without brain injury were enrolled from the neonatal intensive care units (NICUs) of the Massachusetts General Hospital and the Brigham and Women’s Hospital between April 2008 and January 2013. Parents who agreed to participate were asked to read and sign an informed consent form as approved by the Partners Human Research Committee. Infants with gestational age (GA) of 24–28 weeks at birth were eligible for the study. Exclusion criteria included congenital brain malformation; genetic disorders; metabolic or structural abnormality or neoplasm; congenital heart disease; congenital hydrocephalus; imaging evidence of brain lesions other than GM-IVH or white matter abnormalities; or brain infection; Infants with cystic periventricular leukomalacia (PVL) were also not included because the pathogenesis of cystic PVL might affect cerebral oxygenation in ways different from GM-IVH^55^.

### Routine head ultrasound

All of the premature infants had at least three serial HUS for general screening per NICU protocol at three time points: Day of Life (DOL) 1–4, DOL 7–10, and DOL 30. Additional HUS scans were performed as needed, depending on the infants’ clinical conditions. We first screened GM-IVH infants based on the reports of on-site radiologists and/or neonatologists at each hospital. Due to the low agreement in assessments of HUS in detecting low-grade GM-IVH^7^,^8^, all the HUS images were examined and interpreted again by an experienced neonatal neuroradiologist (PEG) blinded to the results of FDNIRS-DCS measurements. The Papile classification was used to grade the severity of GM-IVH on ultrasound^56^.

### Near-Infrared Spectroscopy measurements

We used customized frequency-domain near-infrared spectroscopy (FDNIRS) system (Oxiplex, ISS, Inc. Champaign, IL) and an in-house built diffuse correlation spectroscopy (DCS) system to quantify hemoglobin concentrations (HbO & HbR) and direct measures of an index of cerebral blood flow (CBF_i_). The details of the underlying optical theory and instrumental technology are described in previous work^31^. In brief, the FDNIRS device has two identical measurement modules consisting of eight laser sources of different wavelengths ranging from 660 to 830 nm and two detectors. Four source-detector distances are used in the multi-distance method to determine both scattering and absorption coefficients^52^ and thus absolute hemoglobin concentrations and oxygenation are quantified.

Although DCS measures of CBF_i_ are relatively new, these measures have been extensively validated against various conventional CBF measurement modalities^26^,^27^,^28^. DCS measures microvascular blood flow in cortical tissue by quantifying temporal intensity fluctuations after light has been scattered many times by moving red blood cells^57^,^58^. DCS is similar to laser Doppler blood flowmetry (they are Fourier transform analogs), but in DCS the source and detectors are spatially separated by a few centimeters. Thus, the DCS signal is averaged over a greater cortical volume and is more weighted towards capillary flow than laser Doppler. Accordingly, DCS has been found to be a more consistent measure of cortical blood flow^59^. Together, FDNIRS and DCS are combined to make direct quantitative bedside measurement of not only SO_2_, but also CBF_i_, from which a cerebral oxygen metabolism index (CMRO_2i_) can be determined.

We have designed our own FDNIRS-DCS probe specifically for preterm neonates and have fabricated it in house by 3D printing. The source-detector separations of 1 to 2.5 cm yield a depth penetration of about 0.5–1.5 cm, which mostly includes the cerebral cortex in premature neonates^60^. For measurements, the probe is gently placed on the infant’s scalp in several locations, covering left, middle and right frontal areas^32^, and measurements are repeated several times to ensure reproducibility of the results. The probe is repositioned in a slightly different area to compensate for local inhomogeneities such as hair and larger superficial vessels, ensuring measurements are representative of the underlying brain region rather than circumstantial artifacts. Once the clinical condition of a participating infant permitted, we would measure infants up to once a week during their hospital stay. In addition, the arterial oxygenation (SaO_2_) was recorded from the clinical oximeter at the infant’s bedside at the time of the measurement and hemoglobin in the blood (HGB) was obtained from clinical charts in order to estimate CMRO_2i_^31^,^53^. We have adopted this approach^61^ in over 300 infants in the NICU environment^30^,^31^,^32^.

### NIRS Data Analysis

We have developed our own MATLAB scripts which automatically and systematically assess FDNIRS and DCS data quality using previously established statistical criteria^29^. For each subject, each measurement session, and each location, we calculated absolute hemoglobin concentrations using the FDNIRS multi-distance method^62^. The total hemoglobin concentration (HbT) was used to calculate SO_2_ (SO_2_ = HbO/HbT). We obtained a blood flow index (CBF_i_) once per second from DCS^63^. By combining all measured parameters, we can estimate CMRO_2i_ (CMRO_2i_ = CBF_i_ × HGB × (SaO_2_ − SO_2_))^29^.

### Statistical analysis

Only data that passed standard data quality assessment were used for the statistical analysis. We averaged SO_2_, CBF_i_, and CMRO_2i_ from three locations to represent the frontal region. For group difference analysis, we employed multivariate mixed-model regression analyses with NIRS parameters modeled as a four-dimensional Gaussian random effect with an unstructured covariance matrix and unknown mean vector. We also compared models using Bayesian information criterion (BIC) to best estimate time trajectories of each NIRS parameter as a function of PMA or age. Lastly, for each parameter, PMA or age, group and their interactions, and interactions with NIRS parameters were modeled as fixed effects to assess the statistical significance of between-group differences of time trajectories. This model considered the variances of the unequal number of measurement sessions of each subject as well as the total number of sessions for each group. The analysis was performed in the R statistical package (version 3.1.0), using the “lme4”^64^ and “lmerTest” libraries^65^. The clinical data of the two groups were tested by Mann-Whitney U test, X^2^, or t test (as appropriate).

## Additional Information

**How to cite this article**: Lin, P.-Y. *et al*. Reduced cerebral blood flow and oxygen metabolism in extremely preterm neonates with low-grade germinal matrix-intraventricular hemorrhage. *Sci. Rep.* **6**, 25903; doi: 10.1038/srep25903 (2016).

## Figures and Tables

**Table 1:** Clinical characteristics of two groups.

variables	GM-IVH	Control	P-value
N	7	13	
Male	5	9	0.918
Gestational age (wk)♭	25.2 ± 0.32	26.4 ± 0.36	0.015*
PMA at measurement session(wk)§	33.9 (28.7–39.7)	33.1 (27.5–39.9)	0.151
Birth weight (g)♭	901 ± 60	932 ± 71	0.363
Apgar at 5 min§	8 (6–9)	8 (7–9)	0.653
Multiple birth	3	8	0.423
PDA	4	8	0.848
RDS	3	5	0.848
AOP	1	3	0.639
#of measurements	40	83	

AOP (apnea of prematurity), PDA (patent ductus arteriosus) RDS (respiratory distress syndrome).

Data are shown as number (percentage) except when marked as follows:

^♭^Mean ± SE.

^§^Median (range).

^*^Indicated P < 0.05.
